# Spinal Cord Herniation After Posterior Cervical Spine Surgery in a Middle-Aged Man

**DOI:** 10.7759/cureus.85362

**Published:** 2025-06-04

**Authors:** Takahiro Hirano, Masaya Sekimizu, Toshiyuki Shirahata, Taiki Yasukawa, Yoshifumi Kudo

**Affiliations:** 1 Orthopedic Surgery, Showa University School of Medicine, Tokyo, JPN; 2 Orthopedic Surgery, Showa University Koto Toyosu Hospital, Tokyo, JPN

**Keywords:** brown-sequard syndrome, cervical spine surgery complication, late presentation of dural tear, ossification of the posterior longitudinal ligament (opll), spinal cord herniation

## Abstract

Spinal cord herniation is a rare condition arising from idiopathic or traumatic dural defects or fragility. Here we present a case of early-onset spinal cord herniation following cervical posterior decompression and fusion surgery for ossification of the posterior longitudinal ligament (OPLL). The patient, a 55-year-old male, presented with upper and lower extremity weakness and gait disturbance. Cervical spine MRI and CT scans revealed spinal canal stenosis due to OPLL at the C3-C5 level, leading to a diagnosis of cervical OPLL. The patient subsequently underwent a C3-C5 laminectomy, C2-C7 posterior spinal fusion, and C2 dome-like laminectomy. No intraoperative dural injury was identified, and postoperative drainage did not indicate cerebrospinal fluid (CSF) leakage. Immediately after surgery, the patient exhibited impaired deep sensation in the left upper and lower extremities, along with deficits in fine motor function. A cervical spine MRI performed on postoperative day 19 showed spinal cord deviation and strangulation at the C3-C4 level within the epidural space, leading to a diagnosis of spinal cord herniation. The patient underwent urgent surgical intervention on the same day. Intraoperative findings revealed posterior deviation of the spinal cord through a dural defect resulting in significant strangulation. A dural incision was extended at the site of injury, allowing for spontaneous repositioning of the spinal cord, followed by dural repair through suturing. Postoperatively, the patient exhibited marked improvement in deep sensation and regained ambulatory function. Although mild fine motor impairment persists, the patient was able to walk independently, including climbing stairs, and now continues outpatient follow-up. In this case, no evident intraoperative dural injury was observed; however, the onset was presumed to have occurred immediately after surgery. Particularly in young to middle-aged men, this condition should be considered if symptoms worsen, even in the absence of obvious dural injury or CSF leakage, and early imaging studies should be performed.

## Introduction

Spinal cord herniation is a rare condition that is pathologically classified as idiopathic or traumatic, depending on the underlying cause of dural disruption. Furthermore, postoperative spinal cord herniation following surgery is an exceptionally rare complication [1]. In 1973, Cobb et al. reported the first documented case of postoperative spinal cord herniation [2], and some reports have been published. Previous studies showed most cases of postoperative spinal cord herniation occurred more than six months after posterior cervical spine surgery and intraoperative dural tear was identified; in addition, all cases involved young to middle-aged men [3-6].

However, to date, no cases of postoperative spinal cord herniation after posterior cervical spine surgery occurring immediately after surgery have been reported, nor have there been reports of them in the absence of an intraoperative dural tear. In this case report, we presented a case of early-onset postoperative spinal cord herniation after posterior cervical surgery in a 55-year-old male for ossification of the cervical posterior longitudinal ligament (OPLL). In this case, we underscore that early recognition and intervention are crucial for preventing long-term neurological impairment.

## Case presentation

A 55-year-old male with no significant medical history presented to our Neurology Department with a chief complaint of muscle weakness in the left upper extremity and walking disturbance for the past month. Imaging studies, including cervical spine CT scans and MRI, revealed OPLL and severe cervical spinal cord compression, leading to a referral to our department.

**Figure 1:**
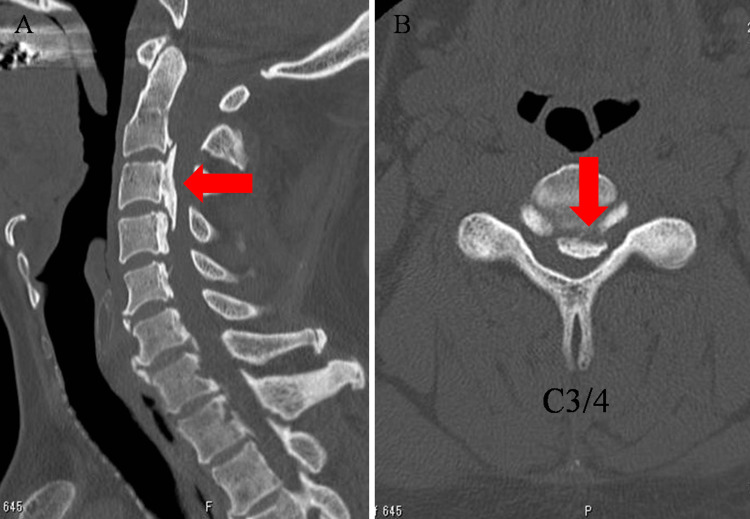
CT scans of the cervical spine at the initial examination (A) CT scan image of the midsagittal section; (B) CT scan image of the horizontal section. Ossification of the posterior longitudinal ligament is seen between C2 and C4 (red arrows).

**Figure 2:**
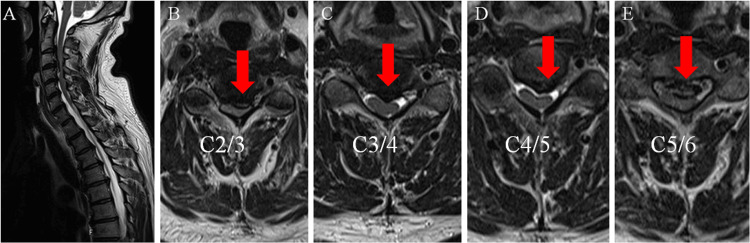
MRI of the cervical spine at the initial examination (A) MRI of the midsagittal section; (B-E) MRI of the horizontal section. Spinal canal stenosis is seen at C2/3, C3/4, C4/5, and C5/6 (red arrows).

On physical examination, the patient presented with deltoid and biceps muscle weakness in the left upper extremity, while muscle strength remained intact in other muscles. The sensory assessment indicated diminished temperature and tactile sensation within the T6-T11 dermatomes, along with reduced vibratory sensation, primarily in the lower extremities. Exaggerated tendon reflexes were observed bilaterally below the biceps with no abnormal reflexes. The patient experienced difficulty with fine motor skills, such as dropping chopsticks and dysgraphia. He was able to walk on level ground without any assistance; however, he needed a handrail to climb stairs. Imaging findings revealed OPLLatC2-4 on the cervical spine CT scan images (Figure 1) and spinal canal stenosis at C2/3 to C5/6 on the cervical spine MRI (Figure 2).

**Figure 3:**
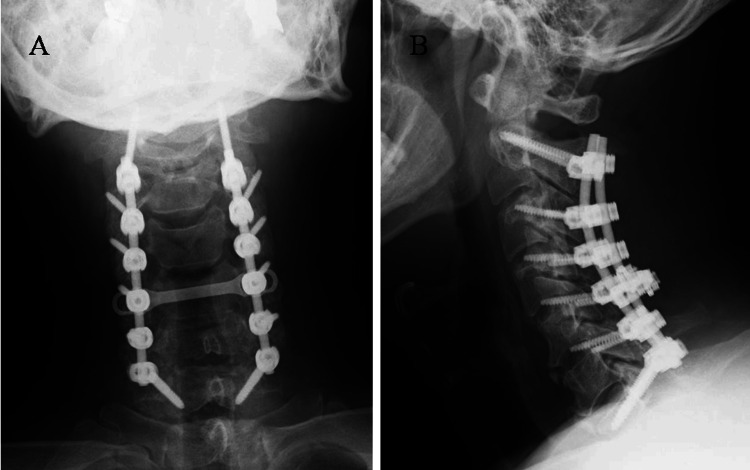
X-ray after cervical decompression and fusion surgery (A) Frontal view; (B) Lateral view. C3-C5 laminectomy, C2-C7 posterior fusion, and dome-like laminectomy of C2 were performed.

A diagnosis of cervical myelopathy associated with OPLL was determined based on clinical presentation and imaging findings. Due to the patient’s impaired fine motor skills and gait disturbance, the patient subsequently underwent a C3-C5 laminectomy, C2-C7 posterior spinal fusion, and C2 dome-like laminectomy (Figure 3). Posterior decompression with fusion was chosen after a shared decision-making process with the patient. During the surgery, the C3-5 laminectomy was performed prior to the C2 dome-like laminectomy, which allowed for improved visualization and access to the caudal aspect of the C2 lamina. A high-speed drill was used to thin the C2 laminae down to the ventral cortical layer. In regions where the drill tip could not be fully visualized during deep bone removal, we preserved the superficial cortex and used Kerrison Rongeurs to complete the excision. Although some steps in a dome-like laminectomy inherently involve temporary limitations in visualization due to anatomical constraints, all maneuvers were performed with maximal attention to safety and visibility. Subsequently, the surgical field was carefully inspected to ensure there was no evidence of dural injury or CSF leakage, and the procedure was concluded.

**Figure 4:**
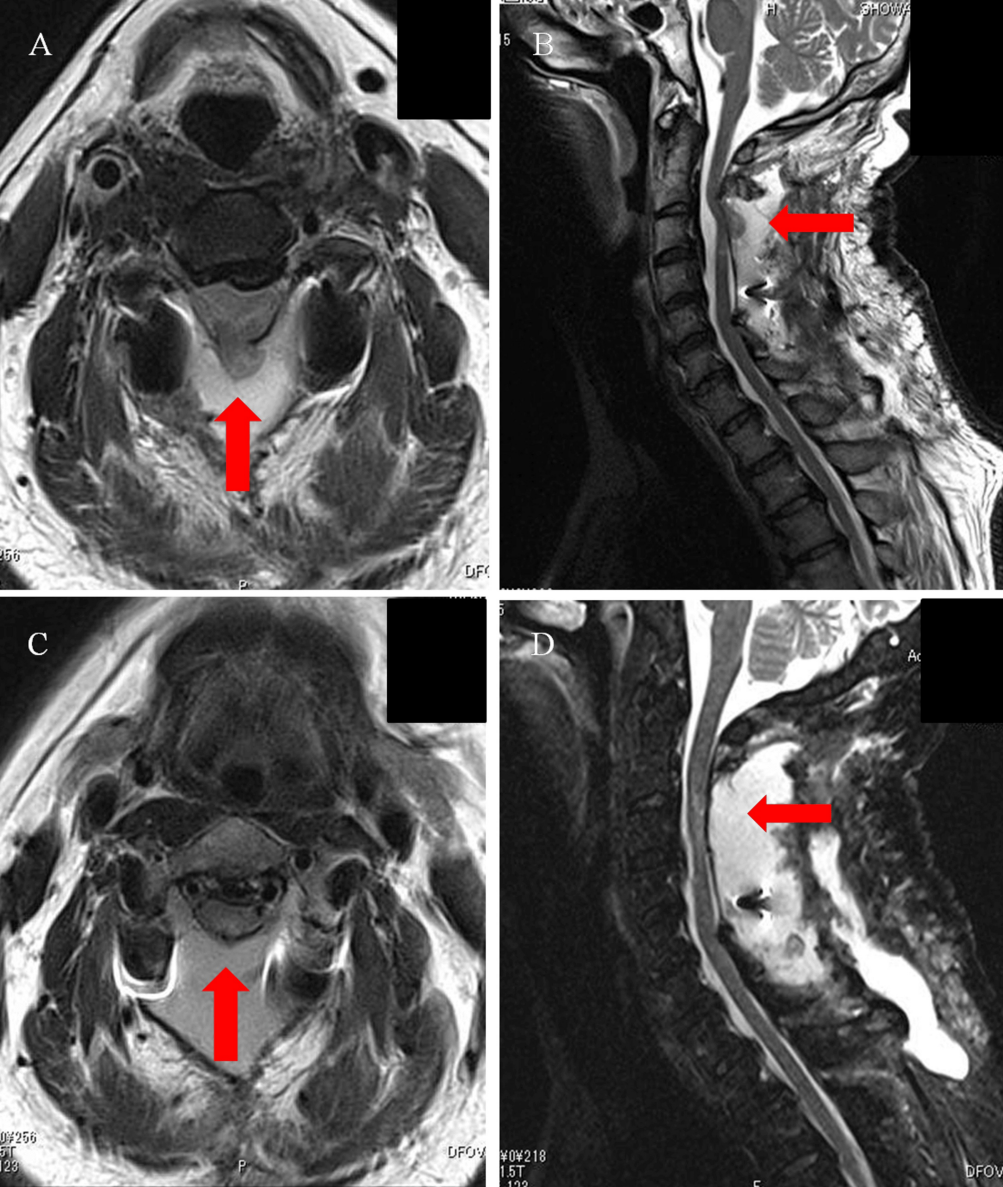
Postoperative MRI T2-weighted images of the cervical spine and postoperative MRI T2-weighted images of the spinal hernia return (A) Sagittal section and (B) horizontal section showing a deviation of the spinal cord (red arrows) from the dorsal interdural space at the C2/3 level. (C) Sagittal section and (D) horizontal section demonstrating retraction of the spinal cord hernias shown in (A) and (B) (red arrows).

**Figure 5:**
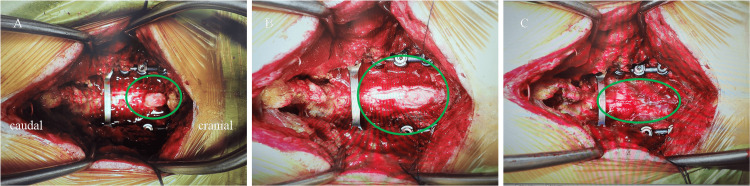
Intraoperative image of spinal hernia repositioning (A) Deviation of the spinal cord from the injured dura mater (oval). (B) Return of the spinal cord through an extended incision from the caudal side of the injured dura mater (oval). (C) Suturing of the dura mater with a 6-0 nylon thread (oval).

On postoperative day one, the patient exhibited diminished tactile and positional sensation in the left upper and lower extremities, with a manual muscle testing grade of three and numbness in the left upper extremity distal to the deltoid muscle. A cervical spine CT scan was performed the following day to confirm the positioning of the instrumentation. At this stage, we assumed that the symptoms were attributed to intraoperative stimulation and spinal cord edema secondary to decompression, characteristic of white cord syndrome [7,8]. By postoperative day six, the patient began gait training, and by postoperative day 11, his symptoms had gradually improved, allowing him to stand independently. Over the following days, the symptoms persisted, and despite initial rehabilitation efforts, fine motor skills and gait disturbance showed minimal improvement. Due to these unresolved neurological deficits, further imaging was warranted. A cervical spine MRI was performed to evaluate the postoperative status of the spinal cord on postoperative day 19. The MRI showed spinal cord deviation from the dorsal aspect of dura matter at the C2/3 level (Figure 4A; Figure 4B). This finding was considered highly indicative of spinal cord herniation. Based on these imaging findings and the patient’s persistent symptoms, an immediate reoperation was performed on the same day to reposition the spinal cord and repair the dura mater. During surgery, a dural tear was detected at the C2/3 level, with evident spinal cord herniation (Figure 5A). With the extent of the incision of the dura mater, the reposition of the spinal cord was achieved spontaneously (Figure 5B). The dura mater was subsequently sutured using 6-0 nylon thread, with no apparent CSF leakage confirmed (Figure 5C). Immediately after the surgery, the patient’s symptoms improved markedly, although numbness in the left fingers persisted. MRI performed on the eighth day after reoperation showed a reduction of the deviated spinal cord (Figure 4C; Figure 4D). One month after reoperation, the patient was transferred to a convalescent rehabilitation hospital. The patient was able to walk without any assistance, including climbing stairs six months after the initial surgery. Eventually, his Japanese Orthopedic Association score improved to 15/17 (11/17 before surgery).

## Discussion

Postoperative spinal cord herniation after posterior cervical spine surgery is an even rarer complication, with a reported incidence rate of only 0.04% (2/4477 patients) [1]. To our knowledge, only 10 patients with postoperative spinal cord herniation of the cervical spine have been reported [2-6]. Almost all cases, except for one, experienced new neurological deficits in the late postoperative period, occurring more than six months after surgery. In addition, previous reports have documented all cases of postoperative spinal cord herniation in the cervical spine as delayed complications, typically occurring after the repair of an intraoperative dural tear. However, we encountered an early-onset postoperative spinal cord herniation following cervical spine surgery, with no apparent dural tear identified intraoperatively.

In our case, although the patient exhibited ipsilateral motor weakness and partial sensory impairment, specifically diminished tactile and positional sensation, in the left upper and lower extremities on postoperative day one, there was no definitive contralateral loss of pain or temperature sensation nor complete sensory loss at the affected level. Based on these symptoms, the presentation was considered an incomplete form of Brown-Séquard syndrome. We presume that herniation of the left hemicord resulted in compression of the dorsal and lateral columns on that side, leading to Brown-Séquard-like symptoms. As Groen et al. have reported in idiopathic cases, hemicord compression can produce comparable neurological patterns [9]. Although the underlying pathophysiology differs from that of idiopathic spinal cord herniation, the mode of symptom onset appears similar.

**Figure 6:**
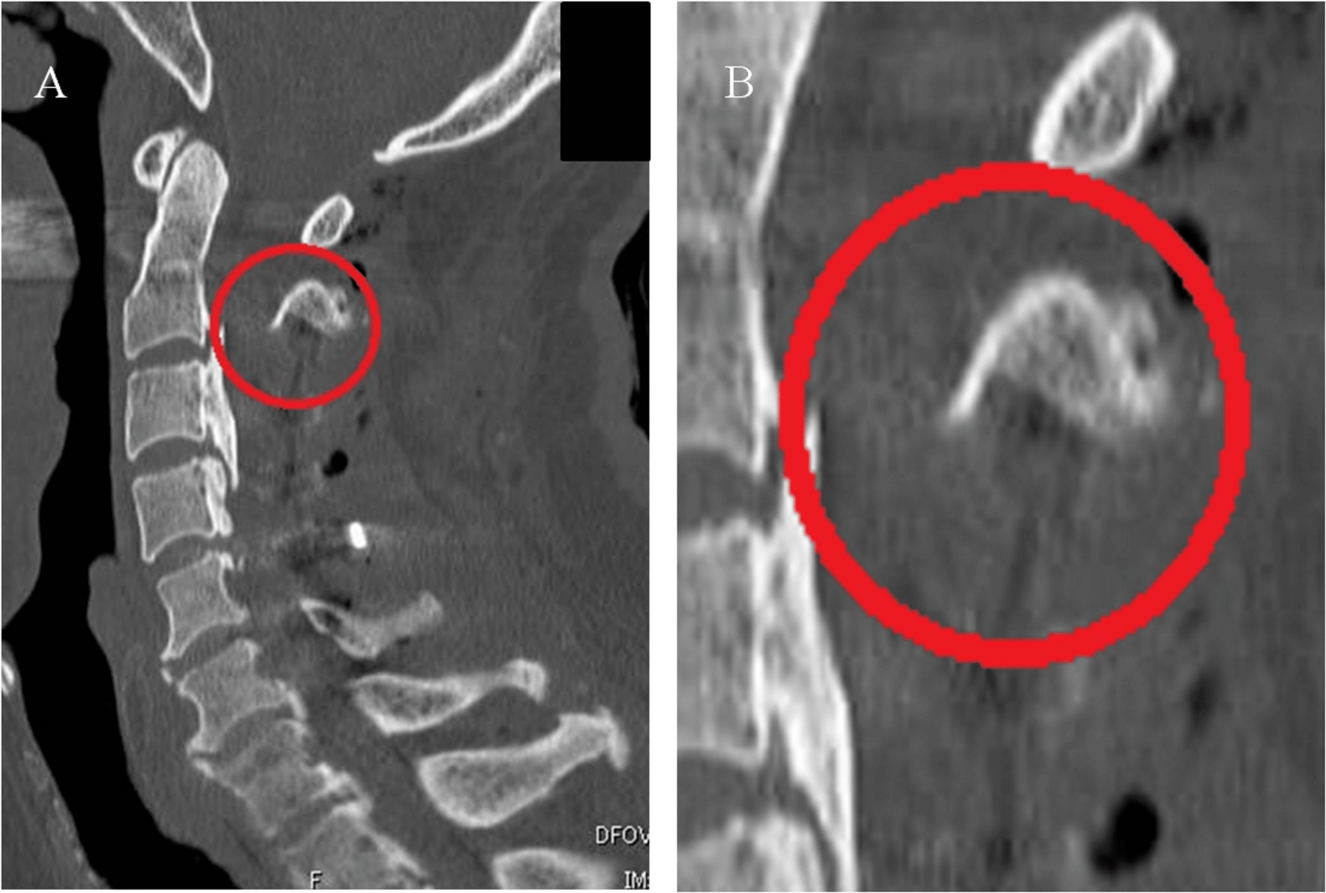
CT images of the cervical spine taken the day after surgery (A) Midsagittal section; (B) Magnified image. C2 dome-like laminectomy was performed on the C2 lamina. The residual edge (ovals) at the base of the C2 lamina were observed, which may have contributed to dural damage, as their location coincided with the site of spinal cord herniation.

As the potential cause of early-onset postoperative spinal cord herniation in this case, we consider the late presentation of dural tear (LPDT) [10-13]. Chang Xu et al. reported that LPDT can result from factors such as decreased dural resistance due to intraoperative dryness, increased CSF pressure from coughing or sneezing, mechanical stress during surgery, or postoperative irritation from bony structures. During the surgery, as described in the Case Presentation, a C2 dome-like laminectomy was performed and there was part of the blind maneuver under the C2 lamina. Postoperative CT showed a residual edge of C2 laminae in contact with the dura mater (Figure 6). Although this edge may appear sharp on sagittal CT slices, they were not considered hazardous intraoperatively and were not intentionally left behind as dangerous projections.

Our hypothesis is that the dural tear was either the consequence of a microscopic injury that was present intraoperatively but undetectable at the time or it occurred postoperatively as a result of mechanical irritation from residual edges exacerbated by excessive dural sac pulsation during extubation. In this context, the present case may be interpreted as a variation of the LPDT mechanism. Further accumulation of similar cases would be valuable in clarifying the underlying mechanisms.

Detecting small dural injuries intraoperatively remains challenging; however, dura expansion tests may aid in their identification. Intraoperative ultrasound technology [14] exists but has not yet been utilized for detecting dural damage. However, future advancements may enable real-time detection. Furthermore, preventive reinforcement using dural sealants or protective barriers in high-risk areas could be considered to minimize the risk of delayed dural injury. In the treatment of spinal cord herniation, surgical intervention is required to reduce the herniated spinal cord and close the dura mater. In previous reports, the repair of the defect required simple enlargement of the dural injury followed by temporary suturing, while in some cases, dural defect enlargement and reconstruction with an artificial dura mater were necessary [15]. In this case, we were able to reduce spinal cord herniation and repair the dural tear easily since a relatively short time had passed after the surgery. Also, the symptoms of this case had improved rapidly after surgery, consistent with findings in other studies.

## Conclusions

We experienced a case of early-onset postoperative spinal cord herniation after posterior cervical spinal surgery. The diagnosis was delayed as the postoperative CT scan revealed no implant placement issues and no evidence of visible dural tear during surgery, leading us to initially conclude that there were no significant complications. However, the patient’s persistent neurological symptoms prompted further imaging, ultimately leading to the diagnosis. This case presentation provides clinicians with the following two important messages: 1) early-onset postoperative spinal cord herniation after cervical spine surgery can occur, even in the absence of a visible intraoperative dural tear, and 2) early recognition and prompt surgical intervention may lead to a favorable outcome. Particularly in young to middle-aged men, careful postoperative monitoring may be essential regardless of the perceived risk of dural injury. Clinicians should maintain a high index of suspicion and consider early MRI evaluation when unexplained neurological deficits arise, as timely reoperation can significantly improve patient outcomes.
